# A Conceptual Framework for Evaluating Impairments in Myasthenia Gravis

**DOI:** 10.1371/journal.pone.0098089

**Published:** 2014-05-20

**Authors:** Carolina Barnett, Vera Bril, Moira Kapral, Abhaya Kulkarni, Aileen M. Davis

**Affiliations:** 1 Division of Neurology - Department of Medicine, University of Toronto and University Health Network, Toronto, Canada; 2 Institute of Health Policy, Management and Evaluation, University of Toronto, Toronto, Canada; 3 Department of Medicine, University of Toronto and University Health Network, Toronto, Canada; 4 Department of Neurosurgery, Hospital for Sick Children, Toronto, Canada; 5 Division of Health Care and Outcomes Research, Toronto Western Research Institute, University Health Network, Toronto, Canada; 6 Department of Physical Therapy and Graduate Department of Rehabilitation Science, University of Toronto, Toronto, Canada; Istanbul University, Turkey

## Abstract

**Background:**

Myasthenia gravis is characterized by weakness and fatigability of different muscle groups, including ocular, bulbar and the limbs. Therefore, a measure of disease severity at the impairment level in myasthenia needs to reflect all the relevant impairments, as well as their variations with activity and fatigue. We conducted a qualitative study of patients with myasthenia, to explore their experiences and related impairments, aimed at developing a conceptual framework of disease severity at the impairment level in myasthenia gravis.

**Methods:**

Twenty patients representing the spectrum of disease participated in semi-structured interviews. Interviews were recorded and the transcripts were analyzed by content analysis using an inductive approach with line-by-line open coding. Themes were generated from these codes.

**Results:**

Two main themes were identified: the severity of the impairments and fatigability (i.e., triggering or worsening of an impairment with activity). The impairments were further classified within body regions (ocular, bulbar and axial/limbs). Fatigability was described as a phenomenon affecting the whole body but also affecting specific impairments, and was associated with fluctuation of the symptoms. Patients were concerned that clinical examination at a single point in time might not reflect their true clinical state due to fatigability and fluctuations in severity.

**Conclusions:**

This conceptual framework reflects the relevance of both severity and fatigability in understanding impairment-based disease severity in myasthenia. This framework could inform the development of impairment measures in myasthenia gravis.

## Introduction

Development of outcome measures begins with a thorough understanding of the concept that is being measured. This is not always simple since many clinically relevant outcomes represent complex phenomena. Including items that are not relevant to the construct of interest or worse, omitting relevant ones can undermine content validity [1]. The International Classification of Functioning, Disability and Health (ICF) [2] defines impairments as significant deviations or loss of body functions (e.g. muscle power, speaking, seeing, etc.) or body structures (e.g. arms, legs, eyes). In the case of Myasthenia Gravis (MG) it has been shown that, based on the ICF definition, impairments of body structures and function are most strongly correlated with the difficulties encountered by patients with MG in their daily life [3]. Therefore, quantifying impairment can provide a measure of disease severity.

The underlying defect of neuromuscular transmission in MG is manifested clinically by muscle weakness and fatigability, which can improve with rest and frequently results in fluctuation of symptoms [4]. MG can cause impairment of extra-ocular, bulbar, axial and limb muscles and these are variably affected in different patients, such that some have purely ocular disease while others have different combinations of bulbar, limb and ocular impairments [4], [5]. All of these factors make measuring impairment challenging in MG patients. Currently available impairment measures in MG were mostly developed based on experts' consensus of relevant impairments [6]–[9]. These measures differ in both the impairments included and how they are measured demonstrating that there is no global consensus. Incorporating the patient's perspective provides invaluable information regarding which impairments are most relevant to patients as well as the patterns of impairment experienced by patients. While some of this information in MG has been gathered through patient surveys [10], or through structured interviews using the ICF checklist of impairments [3] these methods do not allow the depth of inquiry needed to understand complex phenomena. Furthermore, the use of structured response options doesn't easily allow for the incorporation of individual patients' experiences. Finally, some have found low reliability for the ICF codes used in some surveys [11]. Given these gaps in the literature, the aim of this study was to explore the experiences of patients with MG, specifically those related to their impairments, to guide the development of a new impairment measure in myasthenia.

## Methods

### Study Design

This qualitative study used in-depth interviews with content analysis to explore patient experiences. Content analysis is focused on unique themes that illustrate a given phenomenon, improving the understanding of subjective experiences [12], and thus provides the opportunity to explore the depth of patient experiences by systematically identifying themes and patterns. This approach is in keeping with the guidance document from the US Food and Drug Administration (FDA) [13] and the Consensus-based Standards for the selection of health Measurement Instruments (COSMIN) [14], which recommend the incorporation of patient input and encourage the use of a conceptual framework to design new patient reported outcomes (PRO).

### Sampling and Data collection

Adult patients with a confirmed diagnosis of MG attending the Neuromuscular Clinic at Toronto General Hospital (Toronto, Canada), who were fluent in written and verbal English, were invited to participate. Theoretical sampling was used to achieve maximum variation [15]. Hence, patients with varying disease localization (i.e. ocular or generalized) and symptomatic complaints were invited to participate. The Myasthenia Gravis Foundation of America (MGFA) classification [16] was used to classify patients according to their symptom distribution and severity. This classifies patients in class I if purely ocular, and classes II, III, IV,and V for generalized patients with increasing severity. For patients in class II and higher, the subclass “b” indicates primarily bulbar impairments and “a” primarily limb or axial impairments.

There is no consensus on what constitutes a sufficient sample size for qualitative studies [17], however most researchers agree that once new themes are no longer being generated with subsequent interviews (i.e. data saturation), sufficient sample size has been reached for understanding the phenomenon of interest [18]. Therefore, we conducted interviews until data saturation was achieved. The University Health Network Ethics Board approved the study and all patients provided written informed consent.

**Figure 1:**
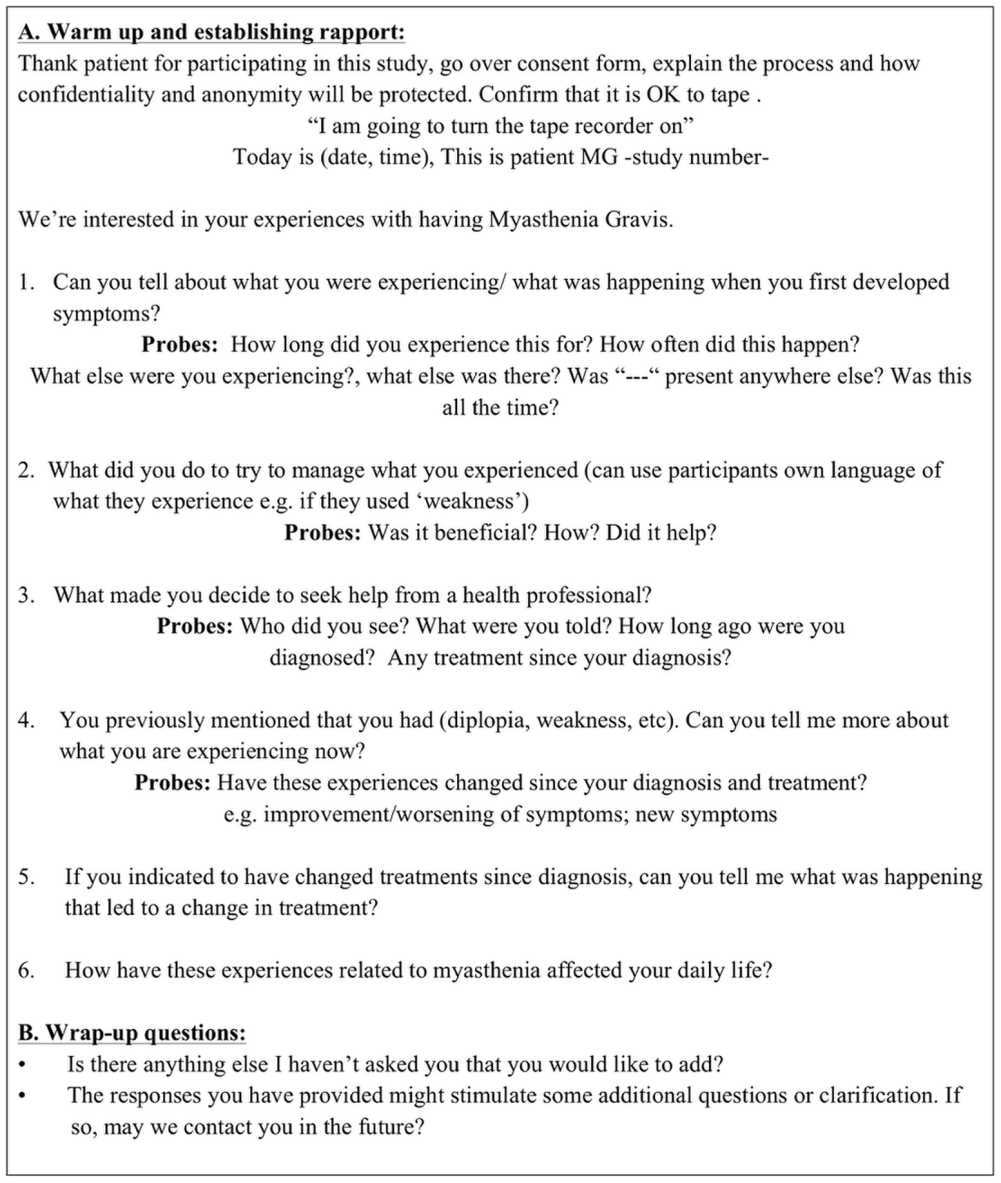
Interview Guide. This is the interview guide used for the interviews. It depicts the open questions and possible probes.

Individual interviews were conducted following a semi-structured interview guide (Figure 1). Interviews were chosen as they allow in-depth exploration of individual participant's experiences whereas focus groups rely more on group interaction to elicit information [18].The interviews began with broad, open questions regarding the patients' experiences with MG with probes used to facilitate more in-depth description. One author (CB) conducted all patient interviews after receiving training in interviewing techniques and performing pilot interviews using the interview guide. The interviews were conducted in person, audio taped and transcribed verbatim. Additionally, memos were created after each interview to allow for interviewer reflection and consideration of any bias that might have influenced the interview. The transcripts were imported into HyperRESEARCH (version 3.5, Researchware, Inc) software for managing qualitative data. The transcripts were analyzed using content analysis which is a process whereby the data are systematically analyzed by classifying words or phrases that have the same meaning together, aiming to explain the phenomenon of interest through these categories or themes [19].

We used an inductive approach, thus assuming no previous knowledge of the phenomenon and used line-by-line open coding [19]. Two authors, (CB and AD) independently coded the first 6 transcripts and then discussed the codes to develop a coding framework. Coding then proceeded in a constant comparative manner to allow probing of arising topics, and the coding was compared to reach consensus. A third author (VB) also coded a sample of transcripts to further ensure that all relevant themes were identified. At bi-weekly meetings, two authors (CB and AD) discussed the transcripts and arising themes and revised the coding framework as appropriate. After the main themes were developed from the codes, sub-themes were created by further grouping similar codes, using the ICF classification [2].The ongoing discussions regarding the transcript codes and themes not only allowed the investigators to discuss their biases but importantly to discuss how themes were evolving from the codes and their potential relationships as the framework was developed.

## Results

Twenty patients were interviewed, the median age was 62.5 years (range: 29 to 78); 11 (55%) patients were female and the median disease duration was 7 years (range: 1 to 27). Regarding localization and MGFA severity, 4 (20%) patients had purely ocular disease (MGFA class I). Of the patients with generalized disease, 5(25%) were in MGFA class IIa, 8 (40%) were in class IIb, 1 (5%) in class IIIa and 2 (1%) were type IIIb at the time of the interview. The clinical characteristics of the patients participating in this study are summarized in Table 1.

**Table 1:** Demographic Data.

Demographic Characteristics (n = 20)	n (%)/median(range)
Age (years)	62.5 (29–78)
Females	11(55%)
MGFA Class (at the time of the interview)	
n (%)	
I	4 (20%)
IIa	5 (25%)
IIb	8 (40%)
IIIa	1 (5%)
IIIb	2 (10%)
Disease Duration (years)	7 (1–27)
Previous MG Crisis	3 (15%)
Previous IVIG or PLEX	11 (55%)
Employment Status	
Employed (full-time)	8 (40%)
Employed (part-time)	3 (15%)
Studying	1 (5%)
On Disability	4 (20%)
Retired	4 (20%)

Continuous data are expressed as median and range.

Nominal data are expressed as number and proportion of patients.

MGFA: Myasthenia Gravis Foundation of America.

I: Pure ocular disease II: Mild generalized disease.

III: Moderate generalized disease IV: Severe generalized disease.

a =  predominant limb/axial impairment b =  predominant bulbar impairment.

MG: Myasthenia Gravis.

IVIG: Intravenous Immunoglobulin.

PLEX: Plasmapheresis.

**Figure 2:**
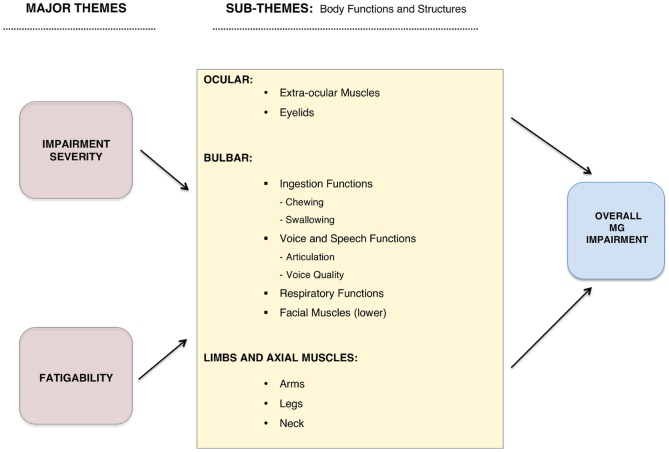
Conceptual Framework for Evaluating Impairments in Myasthenia Gravis. This diagram depicts our proposed framework of disease severity at the impairment level, in myasthenia gravis patients. The main themes (impairment severity and fatigability) were further sub classified by body region, using the ICF classification.

Two main themes were identified that were common across anatomical sites of involvement: the severity of the impairments and fatigability of the impairments (i.e. change or triggering of an impairment with usual activities or onset/worsening of an impairment over the course of the day). Impairments were grouped in 3 sub-themes, based on their anatomical location: ocular, bulbar and axial/limbs. As shown in Figure 2, the impairments were further organized based on the ICF classification of body functions and structures within the sub-themes. The results below have been organized presenting an overview of each main theme with examples for each sub-theme.

### Main Theme: Impairment Severity

Impairment severity refers to the variable extent to which a given impairment was experienced by the patients. The patients used different language to described different impairments within each sub-theme (additional quotes for this theme can be found in Table 2).

**Table 2:** Additional Quotes for Theme: Impairment Severity.

QUOTES
**OCULAR**
**A**. “I began to notice that was the activity [road biking] that required the most intense use of peripheral vision and that was where I first noticed that I was seeing things double.” P3, male, age 66.
**B**. “My eyes will droop so much, that I wouldn't be able to see very clearly.” P17, male, age 61.
**BULBAR**
**A**. “Just a lot of difficulty in chewing meat, then it would get into the point of even chewing something like a soft-boiled egg.” P20, female, age 44.
B. “I couldn't even swallow my pills because I couldn't get the water down.” P14, male, age 64.
**C**. “Every time I drank water, liquids, I always cough… some of the particles that I've been swallowing it goes to my lungs. That's why I have recurrent infection.” P12, female, age 34.
**D**. “And that started with the nasal talking which I'm kind of experiencing a bit today” P5, female, age 33.
**E**. “So I couldn't talk, and sometimes I was talking not clear.” P9, female, age 62.
**F**. “They told me to come back but I told them this is really serious, I really can't breathe anymore.” [Before being admitted to the ICU]. P12, female, age 34.
**G**. “…It was primarily in my face, my forehead. Initially, I guess it's the platysma, the muscle under your skin, it felt like it was very tight.” P7, male, age 63.
**H**. “And the smile, the smile definitely, it's almost like there's no control of the mouth muscles, so when I smile, it's very distorted.” P20, female, age 44.
**LIMBS/AXIAL**
**A**. “The strength in my arms wasn't there anymore, like a lot of times my wife had to put my t-shirts on, I couldn't put my hands above my head.” P4, male, age 60.
**B**. “My arms wouldn't hold any power, like, I didn't have no power.” P19, male, age 63.
**C**. “And it was my feet, my knees and I was working at the bank at the time and I was leaving work and I felt weak on my legs all day…My legs could not hold me.” P5, female, age 33.
**D**. “With the weakness in my neck, I get a lot of headaches because it feels like I have to hold it, to hold it up from it wanting to fall back. It feels weak.” P20, female, age 44.

#### Ocular

In the case of ocular impairments, most of the patients reported double vision. “I *was driving and all of a sudden, instead of one car coming at me there were two coming at me, one in my lane.”* (P8, female, age 63). Some patients described a range of double vision, regarding the separation of the images: “…*it varies from being very little off, so things just almost look a little blurry, to now, I can see two TVs sitting side by side.”* (P11, male, age 78).

The patients also reported drooping of the eyelids and difficulty opening their eyes, such as: *“…there is something weird with my eye, it doesn't appear to want to stay open…”* (P10, female, age 70) and in some cases, they related their most severe experience of eyelid drooping “…*I had to keep my eyelid open with my finger. They wouldn't stay open far enough to actually see.”* (P7, male, age 63).

#### Bulbar

The patients reported different impairments that were classified as bulbar, including problems while eating, speaking, breathing and problems with their facial muscles and expression.

Regarding ingestion functions, patients reported difficulties with chewing, especially with harder foods: *“It's changed dramatically from going to the point where I could not have any kind of solids, … to the point where I could eat anything semi-solid. I can't really eat a steak per se okay or anything like chew celery or chew a raw carrot or a raw apple, that's still difficult.”* (P2, male, age 58). Swallowing was also affected, patients reported different degrees of impairment from choking to difficulty swallowing certain foods: *“…if something goes down the wrong way, I choke fast… before I thought nothing of it, but when I was chewing meat, I was having problems swallowing.”* (P14, male, age 64). Some patients reported difficulties with swallowing fluids: *“I couldn't swallow water, as soon as I swallowed it, it would come back in my nose”* (P4, male, age 60), and some patients had required a feeding tube at some point: *“I wasn't really eating so much… so they put me on a [feeding] tube at home for three years.”* (P12, female, age 34).

The patients also reported two different problems when speaking: problems with the quality of the voice and problems with articulation. The voice quality was affected by low volume or changes in the tone. For example, a participant described the complexity of her voice problems: *“ And then later came my voice. I was getting hoarse, like I am now”* (P1, female, age 39) and from the same patient *“…so I did less speaking and I also used a microphone system, so that amplified my voice…”* indicating not only hoarseness but also low voice volume. Some patients also described difficulties articulating words, such as this patient: *“…I wasn't able to speak, I wasn't able to articulate my words. I literally wasn't able to. I was just mumbling…”* (P2, male, age 58).

Breathing impairments were also frequently reported, sometimes during physical activity: *“…before, when I was walking, I couldn't breathe. But, now it's better.”* (P9, female, age 62). A few patients had experienced a myasthenic crisis: *“…Then two or three days later I was having problems breathing so I came in the hospital, and they put me in a breathing machine because my breathing was very weak.”* (P19, male, age 63).

Some patients reported changes in their facial muscles, in cases producing reduced expression, as this patient describes: *“ [they] were misreading a lot of my facial expressions as being angry or mean because my face wasn't accurately reflecting my emotions. The muscles in my face, I always looked really angry and I also looked sort of tired.”* (P1, female, age 39). Other patients reported impairments localized to their lower face and mouth muscles, for example: *“I couldn't drink from a cup or a straw, it would just run down my face. I had no control [of] muscles in my mouth whatsoever to even seal a straw”* (P20, female, age 44).

#### Limb and axial muscles

The patients reported impairments in their arms, legs and also neck. In the case of the arms, a common impairment was weakness, as reflected in this example: *“…If I were talking on the cell phone too, I wouldn't be able to keep my arm lifted. Sometimes I would have to prop my arm up or something to keep the arm up all the way through [the call]. So, I guess it was the back of the arms. Hard to tie ponytails, hard to shampoo…”* (P6, female, age 29). Some patients described weakness in their hands: *“I would be carrying the mail in from the road and it would just drop out of my hand. I would have a grip on two or three letters and look back and there they were.”* (P8, female, age 63). Participants also described leg weakness, for example: *“I couldn't go upstairs or in the car or the truck or something, I couldn't … my legs, they would just give out, like cooked macaroni.”* (P13, male, age 63). Some patients also described weakness in their neck, with difficulty holding up their head, such as: *“But, honestly, it was so bad that I couldn't hold my head up, every time I talked to somebody my head was kinked down like this”* (P4, male, age 60). The following example illustrates the severity of the neck weakness: *“My head, when I am walking, I have to sometimes keep my chin like that [holds chin with hand], because my head is falling forward. Maybe the muscles in my neck are very, very weak”* (P9, female, age 62).

### Main Theme: Fatigability

Fatigability refers to the triggering or worsening of an impairment with usual or normal activities, or onset/worsening of an impairment over the course of the day. This was reported by all patients and affected different body structures and functions. It was frequently described as weakness occurring right after physical effort: *“Physical labour messes me right up. If I go out to mow the lawn, I'm done for two hours. I have to lie down.”* (P8, female, age 63) And from a different patient, again illustrating the relationship between exertion and the symptoms: *“It improved tremendously and it was almost normal but any exertion was a problem…But as I explained to you, exertion would again bring those things but if no exertion is done then I don't feel it.”* (P16, male, age 64).

Most patients made a distinction between absolute weakness and reduced endurance, as in this example: *“I can climb up the fabric [gym class] and I can do things like that, so I have very strong muscles, which I don't know if that's an indicator or not though because I feel like it's not so much how strong your muscles are, as how intensely you've used them over a long period of time”* (P6, female, age 29). Patients reported having to take frequent breaks, in order to perform tasks: *“And as far as doing anything, I can do anything but I just have to do it inconsistently, like I do a little bit and then I stop for a minute and take a break and do a little bit more.”* (P4, male, age 60).

Participants also described fluctuations of the impairment that reflected fatigability: *“Every time I went home after work, I was so drained. There were times that I was so weak and I couldn't even get up but after having a rest again in the evenings, the day after again when I go to work, I'm okay”* (P12, female, age 34). And from the same patient *“…Some better days, bad days, I don't know, I really can't explain myasthenia. Sometimes I'm good. Sometimes I'm really bad.”* In some patients, these changes were a source of concern regarding their clinical assessments, as this patient indicated: *“It's [the assessment] just such a quick snapshot of how I'm doing, really, at that very moment. And it seems so variable throughout the day. I could have a good hour where people wouldn't even know that I have MG at all. I look like I have lots of energy and whatnot. But then, at a moment's notice, it could completely change.”* (P1, female, age 39). The following also reflects patients concerns during clinical examination: *“I know I'm a lot weaker than what I normally am, what I'm capable of, I know that the muscles are fatigued. But somebody doing the physical test on me, looks at me and says, wow, you've got incredible strength. But it's trying to make sure that the person realises that yes, to you I may look very strong, and I may look like I'm having a great day, but you don't understand, this is not what I necessarily am capable of.”* (P20, female, age 44).

Besides the descriptions of general fatigability above, the patients also described fatigability concerning specific impairments.

#### Ocular

Patients reported that their impairments (eyelid drooping and double vision) could be caused or worsened by prolonged activities with the eyes: *“…And clearly, after a full day of office work, reading, being at my desk, my vision is worse so by the time I go to drive home from downtown [city name] at 5:00 p.m., my vision is probably at its worst that it will be during the day.”* (P3, male, age 66). They also reported fluctuation of the eye impairments, particularly related to the duration of the episodes of double vision: *“Oh, I'll have to say sometimes hours, and sometimes minutes, but then there would be days with nothing.”* (P11, male, age 78). The impact of specific activities and also of the time of day was also reported in drooping of the eyelids: *“… and I would find that by 12:00 in the day time my eyes were like really down. I find out that it is pretty much mostly when I'm tired or especially if I've been staring at something or reading for a bit, then like it [eyelid] really droops.”* (P17, male, age 61)

#### Bulbar

The patients reported a fatigability component with chewing, speaking and breathing. In chewing, fatigability was frequently reported: *“I could chew for a while and then everything stopped, my muscles, I couldn't chew anymore… I'd be getting into a good hamburger and it would just stop,”* (P14, male, age 64) *“I cannot chew gum because one, two or three times if I chew, my jaws I cannot move anymore. I've got to wait 5 or 10 minutes, and then I get a little bit stronger so I gave up chewing gum.”* (P19, male, age 63). But also, some patients reported chewing problems occurring throughout the day: *“I used to have quite difficulty chewing by the end of the day”* (P6, female, age 29).

The patients reported fatigability of their voice triggered by prolonged activity: *“One of the symptoms was that your voice gets tired or whatever… Some people have told me that if I talk for a very long time, there's something in the back of my throat or nasal that starts to click a little bit”* (P6, female, age 29). There were also reports of fatigability of speech: *“I would start sentences and I couldn't finish them. My speech would become garbled by the end of the sentence and it went down from there.”* (P7, male, age 63).

Shortness of breath was triggered by different degrees of exertion: *“…even from the parking lot across the road to come to here, I could do that but I wouldn't be able to come back over and go back across to the parking lot, I would have to stop, it would be too much, my breathing would be affected. I wouldn't be able to go that distance without breathing really heavy, like I've run the Olympics or something.”* (P4, male, age 60).

#### Limbs and axial muscles

There were reports of fatigability in the arms legs and the neck: *“…by the time I wash my hair and brush it and blow dry it, I'm too tired to do what I was supposed to do.”* (P1, female, age 39). For the legs, the patients reported weakness triggered by climbing stairs such as this quote: *“My legs, like I say, if I walk straight it's okay, but if I start climbing stairs they get weak like my knees.”* (P19, male, age 63). Walking was also a reported trigger: *“…after I have walked a little my legs start to feel like lead. It gets harder to pick them up and move them forward.”* (P10, female, age 70). The neck weakness also had a fatigability component: *“…I was working, and maybe three or four hours into my work I am walking, and I had to hold my neck up with my hand.”* (P19, male, age 63). Additional quotes for this theme can be found in Table 3.

**Table 3:** Additional Quotes for Theme: Fatigability.

QUOTES
**OVERALL FATIGABILITY**
**A**. “I find anything that requires endurance is not great for me. But, my muscles are still strong, that's the thing…The muscles are still there. It's not like the muscles have deteriorated.” P6, female, age 29.
**B**. “I honestly thought I was going crazy because I don't know how to explain to you, how do you wake up in the morning and I can say good morning to you, and I could go up the flight of stairs to go to have my shower. And then as the day would progress, originally I would fatigue later on in the day, then it would get sooner and sooner, then it got to the point that it was within an hour.” P20, female, age 44.
**C**. “I think it's the fatigue part. If I've been doing stuff, I get tired. And then, late at night, if I'm watching TV, that's when it usually happens… If we have a function or something, I go, and then by the time I get home, I'm really wiped… But it is a physical tiredness…” P18, female, age 64.
**OCULAR**
**A**. “But as soon as I tried to focus on stuff like TV or reading or watching something for a little bit… that made it really start to go bad. And the more I tried to keep going the worse it got. And the lid would droop…” P10, female, age 70.
**BULBAR**
**A**. “I just couldn't chew enough. I'd wind up with a mouthful of food that was half chewed and I couldn't do anything with it because I couldn't continue to chew…” P7, male, age 63.
**B**. “I could put the food in my mouth, my jaws just would not close, would not come down right and they would fatigue within a minute, two minutes of eating.” P2, male, age 58.
**C**. “And so I would lose it [my voice] for about half an hour and then it would come back…And then eventually it went to the whole morning I'd lose my voice and it would get very, very soft. So I would try to just not talk… And then eventually it went to every day, morning, noon, and night.” P1, female, age 39.
**D**. “At one point I wasn't able to hold a conversation for more than a minute. After that it just became worse and worse. I was more tired.” P2, male, age 58.
**E**. “My speech, when I would first wake up in the morning, was not bad at all, within an hour or two, I couldn't even pronounce words.” P20, female, age 44.
**F**. “Exertion, it will do more… Slight exertion would give you a breathing problem.” P16, male, age 64.
**LIMBS/AXIAL**
**A**. “I couldn't lift heavy stuff. They won't hold. If I lift something maybe for 10 or 15 seconds, then I've got to drop it again. If I have to hold something for a minute or two, I cannot do it.” P19, male, age 63.
**B**. “The legs haven't bothered me for a while unless I intensely exercise them.” P6, female, age 29.
**C**. “I would walk and it was almost like I'd had a stroke. The one foot would come up and just drop.” P8, female, age 63.

## Discussion

This work provides a framework for understanding and evaluating impairments from Myasthenia Gravis based on patient experiences. Patients' descriptions suggest that not only the severity but importantly the fatigability are major drivers of their overall impairment (Figure2). Of current measures, the MG impairment scale [20] has two components: fatigability and strength/function which is in keeping with our two main themes. However, although the specific impairments and their severity related to ocular, bulbar, limb and axial muscles have been incorporated in most MG tools, the inclusion of fatigability has been variable among current measures of impairment [6]–[9], [20]–[23] as shown in table 4. Hence, the main difference between our framework and most current impairment tools in MG is the incorporation of fatigability as a main theme across impairments.

**Table 4:** Characteristics of Current Impairment Tools for Myasthenia Gravis in Relationship with Proposed Framework.

Measure Name	Fatigability Measures Included	Impairments Not Included	Patient Reported Items
QMGS [7], [8]	Endurance: arms, neck, legs	Chewing	None
	Time to diplopia and to ptosis	Voice quality	
	Speech articulation	Lower facial muscles	
MGC [9]	Time to diplopia and to ptosis	Speech articulation and voice†	Chewing
	Chewing	Lower facial muscles	Swallowing
	Breathing		Speech and voice
			Breathing
MMT [23]	None	Chewing	None
		Swallowing	
		Speech articulation	
		Voice Quality	
MMS [6], [30]	Endurance: arms and legs	Ptosis and diplopia†	None
		Speech articulation and voice†	
		Lower facial muscles.	
MG Impairment [20]	Endurance: arms, legs, neck	Speech articulation	Chewing
	Time to ptosis		Swallowing
	Chewing		
	Voice Quality		
	Tongue		
	Swallowing		
MG Score [22]	Arms	Upper and lower facial muscles†	Swallowing
	Legs	Ptosis and diplopia†	
		Speech articulation and voice†	

† Both impairments are combined in a single item.

¶ Unclear whether it is patient reported.

QMGS: Quantitative Myasthenia Gravis Score.

MGC: Myasthenia Gravis Composite.

MMT: Manual Muscle Test.

MMS: Myasthenic Muscle Score.

MG: Myasthenia Gravis.

Fatigability has been defined as exercise-induced reduction in the ability of muscles to produce power [24], or as the magnitude of change in a performance criterion relative to a reference value over a given time of task performance [25]. Fatigability can result in disability if individuals are unable to complete tasks or take longer to do them, limiting daily life activities [24], [25]. Fatigability should be differentiated from fatigue which is a broader concept that includes a mental component [25], and which has been defined as a subjective lack of physical and mental energy that interferes with usual activities [24]. In the case of MG patients, the clinical and electrodiagnostic examinations can provide objective evidence of muscle weakness occurring with activities [4], [5], and this performance fatigability was reported by all our patients. The differentiation between muscle power and fatigability or muscle endurance, can affect how the patients' impairments are perceived when assessed clinically. This might impact clinical decision-making as relevant impairments might be missed by single, fixed point in time measurement without evaluation of fatigability. The result is that some patients might seem to be doing better on a single assessment than their true clinical state measured over the course of their daily activities. Even when the patients used the word fatigue, the probes used to deepen that concept during the interviews usually resulted in descriptions of fatigability, further supporting its importance in understanding impairment in MG.

The importance of the concept of fatigability and reduced endurance was also demonstrated in a study of 102 patients with MG which used the ICF checklist to quantify the prevalence of impairments in body structures and function (and activities and limitations and environmental factors) [26]. The results showed that muscle endurance impairments were more prevalent (77.5%) than impairments of muscle power (54.9%). This further supports our interpretation of our qualitative data.

The reason for the variable inclusion of fatigability in current measures is unclear.

Quantification of fatigability can be challenging and it is possible that is why it has not been widely included in current measures. Although some measures provided limited information on criteria for including and reducing items, in the case of the MGC [9], items were selected from a pool of several available measures used in a clinical trial. Items were chosen based on correlations with quality of life scales and clinical change, such that some endurance items were less responsive and thus were excluded from the final measure. Endurance tests of the arms, legs and neck have been used in some measures as a marker of fatigability, demonstrating responsiveness in clinical trials [27], [28], however, they can be time consuming for routine clinical assessments. Patient reported outcomes asking specifically about impairments triggered or worsened with activity or through the day might be more feasible.

Our framework, as shown in figure 2, includes as sub-themes the following body functions: extra-ocular muscles and the eyelids, ingestion functions (chewing and swallowing), voice and speech functions (articulation and voice quality), respiratory functions, function of facial muscles (lower), and functions of the arms, neck and legs. Currently available measures of impairment have differences among them in terms of the impairments measured as well as with our framework (Table 4). For example, the QMGS [8] includes only swallowing to assess ingestion functions, while the Myasthenia Gravis Composite (MGC) [9] includes swallowing and chewing, in keeping with what our patients reported. Most scales assess speech based on clarity (slurred speech or dysarthria) alone or in combination with tonal changes (nasal voice or hypophonia) within a single item. However, our findings suggest that patients distinguish between tonal and speech articulation impairments such that they represent different phenomena.

Additionally, our findings differ somewhat from those of the study by Leonardi et al. regarding the impairments described [26], and this is likely due to a difference in overall purpose. The aim of the current study was to conceptualize impairments directly caused by MG, as a measure of disease severity. Hence, we did not include impairments that can be caused by other factors such as sleeping functions, which can be secondary to respiratory muscle weakness, medications or depression. This is in contrast to the study by Leonardi [26] that included secondary causes and, using the ICF checklist, found that energy and drive, sleep functions and pain were relevant to patients with MG, in addition to the impairments also present in our framework. While there may be instances where impairments from secondary causes are relevant, we would argue that severity attributable directly to MG more clearly reflects MG impairment.

None of our patients reported eye closure weakness, which is measured in most impairment tools in MG. This is in keeping with the post-intervention status classification by the MGFA, which allows the presence of isolated eye closure weakness as the only clinical sign in patients in remission (no signs or symptoms for more than 1 year) [16]. Together with our findings, this suggests that eye closure weakness is not clinically significant in this population and that it is not informative to measure this impairment in MG patients. In contrast, patients did report lower facial weakness and reduced facial expression, suggesting that those impairments should be measured.

We used a patient-centered approach in developing this framework that will be used to develop a measure of impairment severity. Therefore, it is not surprising that we found some differences compared to current measures given that these were developed mostly based on clinicians' experiences [8], [9], [20], [22], [23], [29], pre-dating current standards for developing patient-reported outcomes that require incorporation of the patient perspective [13].

Incorporating Patient Reported Outcomes (PROs) can be of great value in the case of MG where the impairments fluctuate, as PROs can assess the impairments over longer periods of time than in a typical clinical examination, and thus can be more sensitive to detect clinical change. Further, PROs can assess impairments and their relationship with daily life activities, therefore assessing fatigability, which is harder than with the clinical examination alone. Clinical tests for fatigability typically measure endurance or weakness after repetitive exercise [8], [30], but time constraints might obscure fatigability in patients that require longer activity to trigger their impairments. Therefore combining clinical examination with PROs might be more sensitive to measure overall impairment.

While the qualitative studies are not meant to be generalizable, we do acknowledge that recruitment from a single centre may increase the chances of not identifying relevant impairments. However, while our recruitment site is a large academic centre with more than 300 patients assessed each year, certain experiences such as access to care and treatment patterns and side effects might differ from patients in different settings. Since we were focused on the impairments, which are not related to the treatment environment and are mostly dependent on the patients' individual factors, this might not be an issue. Additionally, we purposely sampled to achieve maximum variation, including a heterogeneous sample of purely ocular and generalized patients with different degrees of severity, to represent the breadth of MG presentation. Further, we did not find any missing themes when looking at the available measures, supporting the main themes and sub-themes incorporated in our framework. Additionally, the investigator who conducted the interviews (CB) had clinical involvement with some of the patients before the interview, and this is a potential source of bias. However, a different investigator (AD), who has no connection to the patients, actively participated in the coding and content analysis, helping to minimize any bias.

In summary, this is the first qualitative study looking at impairments in patients with Myasthenia Gravis. The resulting conceptual framework of disease severity aids to the understanding of the complexity of the impairments, their severity, and fatigability triggered by specific activities and throughout the day. This framework provides the basis for developing new outcome measures or modifying existing ones to better reflect the impairments and symptom burden in patients with Myasthenia Gravis.
